# Effect of zinc on the immune response and production performance of broilers: a meta-analysis

**DOI:** 10.5713/ajas.19.0146

**Published:** 2019-05-28

**Authors:** Cecep Hidayat, Sumiati, Anuraga Jayanegara, Elizabeth Wina

**Affiliations:** 1 Graduate School of Nutrition and Feed Science, Faculty of Animal Science, IPB University, Bogor 16680, Indonesia; 2 Indonesian Research Institute for Animal Production, Ciawi Bogor 16720, Indonesia; 3 Department of Nutrition and Feed Technology, Faculty of Animal Science, IPB University, Bogor 16680, Indonesia

**Keywords:** Zinc, Immune Response, Production Performance, Broiler

## Abstract

**Objective:**

This study performed a meta-analysis of published trials to determine the effects of zinc on the immune response and production performance of broilers.

**Methods:**

A database was built from published literature regarding the addition of zinc forms or doses and their relation to the immune response and production performance of broilers. Different doses or forms of zinc were identified in the database. The recorded parameters were related to the immune response and production performance. The database contained a total of 323 data points from 41 studies that met the criteria. Then, the data were processed for a meta-analysis using a mixed model methodology. The doses or different forms of zinc were considered fixed effects, different studies were treated as random effects, and p-values were used as the model statistics.

**Results:**

An increase in zinc dose increased (p<0.05) pancreas metallothionein (MT) and zinc concentrations in the plasma, tibia and meat, all in quadratic patterns, but linearly decreased (p<0.05) the heterophil/lymphocyte (H/L) ratio. Regarding the different zinc forms, both inorganic and organic zinc increased (p<0.05) the zinc concentrations in the plasma and tibia, the calcium and phosphorus contents in the tibia, and the antioxidant activity of superoxide dismutase in meat as compared to control. An increase in zinc dose increased average daily gain (ADG) and decreased feed conversion ratio (FCR) following a quadratic pattern (p<0.05). Inorganic and organic zinc decreased (p<0.05) FCR and H/L ratio than that of control, but these two forms were similar for these parameters.

**Conclusion:**

Zinc addition has a positive impact on immunity and broiler production. Zinc can suppress stress and inhibit the occurrence of lipid peroxidation in broilers, and it can also improve ADG, FCR, and the quality of broiler carcasses.

## INTRODUCTION

Zinc is an important nutrient required for broilers [1] and plays three important roles in the body that facilitate biological functions: as a catalyst, regulator and structural constituent [2]. Furthermore, zinc is essential since it serves as a cofactor in more than 240 enzymes and helps to metabolize nutrients, such as carbohydrates and proteins, thus helping to increase growth and reproductive performance [3,4]. In broiler diets, zinc may be used either as organic zinc (e.g., Zn protein, Zn amino acid, or Zn picolinate) or inorganic zinc (e.g., ZnCl_2_, ZnSO_4_, or ZnO). The recommended zinc level in broiler diets by the National Research Council (NRC) [5] is 40 mg/kg of diet, which can be supplemented via inorganic or organic forms.

On the other hand, broiler production in tropical countries is generally suboptimal as indicated by the poor growth performance, suppressed immune function, respiratory disease incidence and high mortality rate [6,7], apparently due to the high ambient temperature and relative humidity occurring in the regions. It had been reported that addition of zinc to the diet of broilers reared under heat stress improved the production performance and reduced the feed conversion ratio (FCR) [8,9]. This indicates that zinc addition may be more important for broiler production in the tropics in order to minimize the negative effects associated with such high temperature condition.

Zinc improves the immunity of broilers, and several studies have reported that zinc can improve immunity. First, zinc functions as a cofactor of thymulin and induces proliferation and modulates cytokine release [10]. Second, Sajadifar et al [11] reported that zinc has a role as a nonpharmacologic booster of immunity in broiler chicks. Third, zinc acts as an immunostimulator that is able to enhance both the cellular and humoral immune systems [12]. The role of zinc as an enhancer of broiler immunity is necessary because of the number of restrictions on the use of antibiotics in the diet imposed by many countries. However, the doses of zinc needed to improve the broiler immune response differ across reports, with some studies reporting that the required dose is greater than the NRC [5] recommendation and that zinc has the ability to enhance antibody production. Accordingly, the aim of the current study was to determine the effect of zinc addition to broiler diets on the immune response and production performance through a meta-analysis using data from published journal articles.

## MATERIALS AND METHODS

### Development of the database

A database was constructed based on data from published articles that reported the addition of various doses or forms of zinc to broiler diets and their effect on the immune response. Journal articles were searched using the tools Scopus, Science Direct, and Google Scholar and the keywords “zinc”, “broiler” and/or “immune”. If the papers contained immune response and production performance, the data on production performance would be collected for analysis. However, if the papers only reported production performance without data on immune response, these papers would not be included in the analysis.

The parameters included in the database were weight of the lymphoid organs, zinc concentration in tissue, average daily gain (ADG), feed intake, FCR, mortality, minerals on tibia, blood parameters, haematology, metallothionein (MT), antioxidant activity, immunoglobulin parameters (IgA, IgG, IgM) and complement parameters (C3, C4). After searching using the keywords above, 60 articles were found. The next stage was an abstract evaluation, which resulted in 50 articles that could potentially be used. The next process was an evaluation of the entire article, which resulted in 41 articles that could be used. Finally, the data from 41 journal articles were entered into the database (Table 1). Abstract and full article evaluations were carried out by determining whether zinc was added to the diet and whether immunity or production parameters were examined.

As indicated in Table 1, several forms of zinc were applied, such as organic, inorganic and control (no addition). The added doses ranged from 0 (control) to 200 mg/kg of diet. The added zinc dose did not include zinc derived from feedstuffs. In the process of tabulating data into a database, the data for similar variables were converted to the same measurement units, which facilitated further analysis processing.

### Analysis of data

The data included in the database had compatible measurement units and were further processed in a statistical meta-analysis based on a mixed model methodology [52,53]. Different studies were grouped as random effects, and the dose or different forms of zinc were grouped as fixed effects. The present meta-analysis used two types of statistical models to determine whether the predictor variables were continuous or discrete. The statistical model used in this study was based on p-values. The criterion for determining the significance of the effect for each variable was a p-value <0.05. When the p-value was between 0.05 and 0.1, the effect had a tendency to be significant. All statistical analyses were carried out using SAS software version 9.1 [54].

## RESULTS

The effect of dose or different forms of zinc on broiler immune response are shown in Table 2 and 3, respectively. Generally, the dose or zinc form did not have any significant effect on the weight of lymphoid organs (i.e., thymus, bursa of fabricius, spleen) or hematology parameters (i.e., hemoglobin, hematocrit). The zinc dose decreased liver weight by following a quadratic pattern (p<0.05). The zinc dose that gave the lowest liver weight was 107 mg/kg. Increasing zinc dose significantly reduced the heterophil/lymphocyte (H/L) ratio with a quadratic pattern (p<0.05), and both organic and inorganic zinc were effective in reducing the H/L ratio (p<0.05). The dose of zinc that gave the lowest of H/L ratio was 106 mg/kg. Furthermore, the zinc dose and inorganic form increased the pancreas MT (p<0.05). The zinc dose did not affect antioxidant activity (superoxide dismutase [SOD]) in the meat and liver tissues. Furthermore, zinc dose tended to reduce malonaldehyde (MDA) concentrations in the liver (p<0.10). Different zinc forms increased the antioxidant activity of SOD in meat (p<0.05). Organic zinc resulted in the highest SOD activity in meat. In contrast, the SOD activity and MDA concentrations in the liver were not significantly influenced by different zinc forms. Immunoglobulin parameters (IgA, IgG, IgM) and complement parameters (C3, C4) were not significantly affected by the dose or different zinc form.

The effect of dose or form of zinc on broiler production per formance is shown in Table 4 and Table 5, respectively. The production performance of broilers, i.e., ADG and FCR was significantly improved by increasing dose of zinc (p<0.05; Table 4). As the effect was in quadratic response, the optimum dose of zinc that gave the highest ADG and the lowest FCR were 93.37 and 75.72 mg/kg, respectively. Meanwhile, average daily intake, mortality, carcass percentage, and abdominal fat percentage were not significantly affected by increased zinc dose or different zinc forms. Different zinc forms decreased FCR (p<0.05), although differences were not observed among the two forms. In addition, abdominal fat percentage of broilers reduced compared to that of control group but only inorganic zinc that significantly reduced (p<0.05). No difference was found on abdominal fat percentage between any form of zinc. Zinc concentrations in plasma, tibia and meat increased under increased zinc dose (p<0.05). The blood parameters (total protein and albumin) were not significantly influenced by the dose or form of zinc. Different zinc forms increased zinc concentration in the plasma and tibia (p<0.05), with organic and inorganic zinc producing similar concentrations. The optimum dose of zinc addition in broiler diet which resulted in the best zinc concentrations in the plasma, tibia and meat were 125, 116.5, 63.3 mg/kg, respectively. The different zinc forms tended to increase zinc concentration in meat (p<0.1). Increasing zinc dose tended to increase calcium concentration in the tibia. In addition, an increase in zinc dose did not have any effect on the phosphorous content in tibia, while different zinc forms increased calcium and phosphorous contents in the tibia (p<0.05). Differences were not observed in the effects of organic and inorganic zinc on calcium content in the tibia. Organic zinc resulted in the highest phosphorous content in the tibia compared to inorganic form or control treatment.

## DISCUSSION

### Influence of zinc addition on the broiler immune response

The insignificant effects of zinc addition on thymus, bursa of fabricius and spleen could have been a direct result of the nonsignificant effect of the zinc on feed consumption (Tables 4, 5); thus, the supply of nutrients for the development of these organs was not altered. Bartlett and Smith [16] explained that zinc addition to broiler diets did not affect the relative weight of lymphoid organs because zinc consumed by broilers was preferentially used to support the metabolic processes that support growth performance, whereas the use of zinc for the development of organs related to the immune system was minimized. Cui et al [55] stated that zinc has an important role in the broiler immune system, which can be seen from the limited development of either lymphoid organs or the mature population of blood T lymphocytes when zinc deficiency occurs in broilers. In chickens, zinc-deficient birds have characteristic microscopic lesions in their lymphoid organs [56]. Lymphoid organs are part of the structure and function of the immune system in broilers that can protect the body from attack by microorganisms, and zinc is known to have an important role in the immune system of the animal because it is needed in the function, structure, and development of the immune system [57]. Lymphoid tissue in broilers is divided into two groups: the central lymphoid (thymus, bursa) and the peripheral lymphoid (spleen). The two lymphoid tissues are associated with the intestinal mucosa [58]. The bursa of fabricius is the main lymphoid organ in poultry that has an important function in B lymphocyte differentiation [59]. Tanaka et al [60] reported that zinc is required for lymphocyte proliferation. During organ development, the bursa of fabricius in the body of birds reaches its maximum size at the age of 8 to 10 weeks [61]. Kidd et al [57] revealed that the level of zinc addition in the diet of poultry affected the size of the lymphoid organs, which is related to the function of T cells and decreased when the diet was added with low amounts of zinc. The factor that reduced lymphoid organ weight was heat stress in the environment [62]. Studies by Jeurissen [63] or Smith and Hunt [64] reported that the function of the spleen is to support the development of lymphocytes, which have a crucial function in cellular and humoral immunity. As stated by Moller and Erritzoe [65], the mass size of the spleen is closely related to the level of humoral immunity, and a large spleen size will result in high humoral immunity, and vice versa. Increasing zinc level reduced liver weight percentage compared to other lymphoid organs, because transfer of zinc to liver from plasma is 5 to 6 times faster than transfer to other major tissue [66]. Liver weight affected by dietary zinc alteration may be due to the critical role of liver on zinc metabolism [67].

Hematology parameters (i.e., hemoglobin and hemato crit) were not significantly influenced by the zinc dose. This result indicated that zinc addition may not significantly alter blood constituents. The H/L ratio is an index of stress in birds [68]. The doses and different forms of zinc reduced the H/L ratio, and the various zinc forms, i.e., organic and inorganic, had a similar effect on the H/L ratio. The dose of zinc that gave the lowest of H/L ratio was 106 mg/kg. Studies have shown that increased zinc doses have a positive impact on suppressing stress in broilers. Kidd et al [57] showed that adequate zinc is needed to produce lymphocytes normally during development; therefore, zinc deficiency resulted in a reduction in the number of peripheral T cells, decreased T helper cell function and depleted thymocytes in the thymus. Zinc addition supported the optimum development of lymphocytes, which alleviated stress. An increase in the lymphocyte count and decrease in the H/L ratio might be attributed to decreased glucocorticoid secretion [68]. Furthermore, Walsh et al [69] explained that zinc contributes to noncovalent reactions of cytoplasmic components by tyrosine kinase, which is an essential protein in the early stages of lymphocytes activity. Dardenne et al [70] revealed that zinc is a thymulin cofactor that binds with thymic hormone to surface receptors of T lymphocytes and leads to the maturation and activation of these cells. Zinc binds to thymulin by asparagine side chains and hydroxyl groups. Therefore, the addition of zinc to the diet increased thymulin activity, which led to the more appropriate maturation and activity of T lymphocytes [71].

In addition, the dose and inorganic forms of zinc increased pancreas MT. Zinc has an important role in many physiological functions, which includes numerous metalloenzyme systems [72]. Andrews [73] stated that MT is a cysteine-rich protein that has the ability to bind zinc and other heavy metals involved in stress response activities. Researchers have also demonstrated that MT is synthesized in tissues in response to dietary zinc and binds excess zinc, which has a negative impact on the body [21,74]. Moreover, other researchers, i.e., Wedekind et al [75] and Sandoval et al [74], have reported that pancreas MT protein may represent a good indicator of the zinc status of chickens. The optimum dose that produces the highest pancreatic MT was 90.63 mg/kg. Because the only available data were for the control and inorganic groups, the current study could compare only the effects of the inorganic zinc group and the control group.

The zinc dose did not affect antioxidant activity (i.e., SOD in meat and the liver). Zinc is a cofactor for SOD and has an important function in antioxidant systems, i.e., as an inhibitor of the oxidation process by protecting proteins and enzymes and as an inhibitor of the formation of free radicals [76–78]. SOD is widely distributed and protecting various organs and tissues from peroxidation [79]. Similarly, a study by Zago and Oteiza [80] showed that zinc is the main component of Cu/zinc SOD, and SOD has cell defense functions against oxidative stress, which explains why zinc deficiency can lead to the increased production of free radicals [81].

The MDA in the liver tended to decrease as the zinc dose increased, which indicated that an increasing zinc dose tended to inhibit lipid peroxidation (LPO). Tupe et al [82] revealed that the dietary zinc status exerted a powerful influence on the degree of oxidative damage caused by free radicals. MDA is a product of the process of lipid oxidation. Higher MDA values correlate with higher oxidized lipid values. Lipid oxidation is related to antioxidant activity. The role of zinc, which is important in antioxidant activity, is expected to have an impact on the reduction of the lipid oxidation process [80,83]. Concentrations of MDA in the liver were not significantly influenced by the different zinc forms. MDA is an indicator of the occurrence of LPO; hence, MDA can reflect the hepatocyte membrane damage level and LPO in the liver. Prasad and Kucuk [84] explained that as a cofactor of many anti oxidative enzymes, zinc plays a key role in decreasing the production of free radicals. However, Raharjo and Sofos [85] explained that MDA is also widely used as an indicator of the amount of lipid oxidation in meat. In the current study, although not significantly different, the MDA levels in the liver following organic zinc addition were lower than those in the control. These results showed that zinc has the ability to inhibit LPO. Previously, some researchers, i.e., Powell [86] and Prasad and Kucuk [84], reported that zinc reduces MDA, which indicates that zinc functions as an antioxidant agent because zinc reduces LPO in the cell membrane.

Different zinc forms increased the antioxidant activity of SOD in meat. The addition of organic zinc resulted in the highest SOD activity in meat. Zinc can improve this aspect of immunity because zinc is an important part of the process of cell integration, which is involved in the immune response [70]. The important role of zinc in the activity of the immune response is related to the impact of zinc on the antioxidant defense mechanisms in the body [84]. Tate et al [87] stated that zinc increases antioxidant activity by decreasing the production of free radicals because zinc competes with other minerals, such as copper and iron, in binding to cell membranes. Yamaguchy [88] and Gibbs et al [89] reported that Cu/zinc SOD has an important function in reducing free radical production.

The immunoglobulin parameters (IgA, IgG, and IgM) and complement parameters (C3 and C4) were not significantly affected by the dose or different forms of zinc. Immunoglobulin is a protein that has antibody activity. These results were inconsistent with previous reports by Beach et al [90] and Burns [56], who showed that adding zinc to the zinc deficient diet increased the production of poultry antibodies. Furthermore, Feng et al [20] reported an improvement in immunoglobulin levels (IgA, IgM, and IgG) with the dietary replacement of 120 mg/kg of inorganic zinc (ZnSO_4_) with 90 or 120 mg/kg of organic zinc (zinc-glycinate).

In the current study, zinc had a positive impact on reduc ing stress levels in broilers. This observation is evidenced by the ability of zinc to reduce the H/L ratio and increase the pancreas MT. Zinc also tends to reduce MDA levels in the liver, which shows that zinc tends to suppress the occurrence of LPO. Zinc addition did not significantly affect the other immunity variables observed in this study due to variations in the experimental material used, especially changes related to the time or age at sampling and differences in environmental conditions. Accordingly, this study can be used as a reference for broiler farmers, that the addition of zinc in broiler diet can give benefit to improve their broiler health, through increasing broiler immunity. The optimum dose that produces the best pancreatic MT and reduced stress indicated by H/L ratio were 90.63 mg/kg and 106 mg/kg, respectively, which will perform the best broiler immunity.

### Influence of zinc addition on broiler production performance

The production performance of broilers parameters, i.e., ADG and FCR was significantly improved by increases in the zinc dose (Table 4). This result supported the study before that zinc is the main trace mineral involved in the metabolism of carbohydrates, lipids, and proteins [9]. Zinc has function as an enzyme cofactor that helps in the metabolism of nutrients so that they can be used more efficiently. Different zinc forms decreased the FCR, although the various forms did not show differences in their results. The optimum dose of zinc addition in broiler diet which resulted in the best ADG and FCR were 93.37 and 75.72 mg/kg, respectively. The other production performances of broiler, i.e., average daily intake, mortality, and carcass percentage, were not significantly affected by increasing zinc dose or the different forms of zinc (Tables 4, 5). This study in line with several studies which reported that zinc addition on broiler diet had no effect on average daily intake, mortality and carcass percentage [91–93]. In contrast, several studies reported that zinc addition on broiler diet affected average daily intake, mortality and carcass percentage [51,94–96]. The difference in the effect of zinc addition to the diet on broiler production performance is allegedly also caused by differences in the breed of broiler, growth phase, and the presence of ligands in the diet that are able to bind zinc, i.e., phytate, thereby disrupting the absorption of zinc in the body because zinc is bound to and forms complex bonds [97]. The phytate will form complex bonds with minerals which have valences two, i.e., such as Zn, Mn, Ca, etc., which make those mineral undigested in the poultry’s intestine [98]. Phytase supplementation will help release minerals bound with phytate so that can be absorbed by the poultry’s intestine [99,100].

Another positive effect of the addition of different zinc forms to broiler diets was reduced abdominal fat percentage. Inorganic zinc reduced the abdominal fat percentage of broilers compared to that of the control group. No difference was found on abdominal fat percentage between any form of zinc. In the current study, dietary inorganic zinc reduced the abdominal fat percentage of broiler rather than organic zinc, that is because, firstly, organic zinc sources that analyzed in this study were not the same organic compound (i.e., zinc proteinate, zinc lysine, zinc poly amino acid complex, zinc methionine, zinc glycine, zinc-enriched yeast, zinc propionate, zinc glycinate), that variation may contribute to the insignificant fat reduction effect. In terms of inorganic form, mostly zinc source was coming from zinc sulfate. Secondly. the bioavailability of inorganic zinc in monogastric is much lower [101]. This is because in the monogastric gastrointestinal tract, the inorganic zinc combined with phytic acid. Meanwhile, organic zinc which combined with amino-acids did not interact with phytic acid as they are lacking the free divalent cations needed for chelation in the intestine [57,102]. Higher bioavailability of organic zinc leads to higher fat metabolism in the liver, furthermore increasing the lipid synthesis for broilers through controlling the activities and gene expressions of lipogenic enzymes [103,104].

Ferrini et al [105] stated that efforts to reduce fat deposi tion in the chicken body has become one of the main topics of broiler research. This development is because consumers want poultry products that are high quality and meet high health criteria. The high fat content in animal products is known to be an underlying cause of obesity and coronary heart disease [106]. Reducing abdominal fat in broilers is not only a method of reducing the cost of production but also for improving the efficiency of the feed use because abdominal fat is considered waste. In the other words, the addition of organic zinc in broiler diets helps improve the quality of carcasses by decreasing the percentage of abdominal fat.

The zinc concentrations in the plasma and tibia increased with the dose and form of zinc. Organic and inorganic zinc addition produced similar concentrations. An increasing zinc dose and the addition of organic or inorganic zinc increased the zinc concentrations in the plasma and the tibia. The zinc concentrations in the plasma can be used as an indicator of the amount of zinc digested by the body of poultry in certain periods [107,108]. Therefore, the zinc concentrations in plasma are the most widely used indicator of zinc status because low values are considered an early symptom of zinc deficiency [12]. The optimum dose of zinc addition in broiler diet which resulted in the best zinc concentrations in the plasma, tibia and meat were 125, 116.5, and 63.3 mg/kg, respectively. Bone is a mineral storage site, and zinc from the bone can be used by the body when there is a zinc deficiency [16]. The zinc content in bone is an appropriate indicator of zinc bioavailability in poultry diets [18]. The relationship between the zinc dose and zinc concentration in the plasma, tibia and liver followed a quadratic linear pattern with a positive slope for all variables, indicating the bioavailability of zinc [109]. Grynpas et al [110] stated that another reason why the levels of zinc in bone are an indicator of the bioavailability of zinc is because bone is a large storage area for zinc. For example, the zinc content in bone can reach 300 mg/g; thus, zinc is an important element in bone metabolism. Accordingly, an adequate zinc concentration is required for the growth, development and mineralization of bone [84]. Bones become a zinc reserve in the body, and when a zinc deficiency occurs, the zinc in the bones will be used. The metabolism of zinc from bone is very dynamic, and zinc is distributed from the bone to other tissues when the body is deficient in zinc [74,107]. Furthermore, Attia et al [111] stated that zinc concentrations increase in the tibia due to the increased consumption of zinc-supplemented diets and higher subsequent delivery of both supplemental and basal zinc. Moreover, previous studies have demonstrated that the zinc content of the tibia is significantly influenced by dietary concentrations, and Loveridge [112] revealed that the highest zinc concentration in the tibia resulted from addition levels similar to those that resulted in birds attaining an optimal body weight. The mineral content in tissue is often used as an indicator of the mineral status of animals as well as the level of minerals consumed by animals [113]. In the current study, the average dose of organic zinc was 62.83 mg/kg, while that for inorganic zinc was 62.89 mg/kg (Tables 3, 5). In this study, the average dose for organic and inorganic zinc is almost similar, which generally has a similar effect on the immune response and production performance of broiler. Comparing the current price of organic and inorganic zinc. Generally, the price of inorganic zinc cheaper than organic zinc, for example, the price of zinc-methionine for feed (organic zinc) is 5980–6120 USD/metric ton [114], while the price of zinc sulfate for feed (inorganic zinc) is 900–1,100 USD/Metric ton [115]. Accordingly, using inorganic zinc, economically more efficient than organic zinc.

Generally, the effect of the different form of zinc does not have much effect on the parameters of production performance. This study differs from the several reports which stated that organic zinc will be better at giving effect to broiler production because it has better bioavailability. As reported by Berger et al [116] and Salim et al [117] who showed that the bioavailability of organic zinc was shown to be higher than that of inorganic zinc such that the amount of organic zinc used in the diet is less than that of inorganic zinc. The bioavailability of zinc greatly determines how many doses of zinc is used in the broiler diet, the higher bioavailability of zinc, the lower the dose of zinc used in the diet. The dose of zinc used in the broiler diet must be in accordance with the zinc requirement of a broiler because excessive use of zinc will cause zinc not to be used by the broiler body, thereby released through manure [118], which caused environmental pollution. The study by Zhang et al [119] showed that animal manure is an important source of heavy metals to the environment. The study by Giordano et al [120] reported that high concentrations of zinc on water and soil reduced crop yields. It is because zinc increased the acidity of waters so that zinc can interrupt the activity in soils, as it negatively influences the activity of microorganisms and earthworms, thus retarding the breakdown of organic matter. In the future, to suppress environmental pollution from zinc excreted through manure, zinc has been developed in the form of nanoparticles. The development of zinc nanoparticles are intended to reduce the dose of zinc used in the diet, hence, the lower dose of zinc added to the diet, accordingly, will be even less of zinc that excreted through manure. Feng et al [121] stated that zinc nanoparticles are more effective than the larger size of zinc at lower doses.

Increasing the zinc dose tended to increase the calcium concentration in the tibia. The quality of tibias was affected by element supplementation in the diet [94]. These results are consistent with those of Rayani et al [122], who stated that an increase in the zinc dose from 40 to 80 mg/kg significantly increased the calcium content of the tibia bone, with the highest calcium levels of 51.97% found when the experimental broiler chickens were added with 80 mg/kg of zinc. Zinc addition at a dose of 80 mg/kg increased the calcium content by up to 37.41% [25]. In addition, increasing the zinc dose had no effect on the phosphorus contents in the tibia. The current study differs from that of Sunder et al [25] and Vieira et al [26], who showed that zinc addition significantly affected the phosphorus contents of the tibia. Different zinc forms increased the calcium and phosphorous contents in the tibia, although the effects of organic and inorganic zinc on the calcium content in the tibia did not differ. Organic zinc induced the highest phosphorus content in the tibia, thus indicating that organic zinc was efficient at supporting calcium and phosphorus deposition in the tibia. This result suggested that organic zinc was efficient at supporting calcium and phosphorus deposition in the tibia, indicating that various zinc forms did not have an antagonistic effect on the deposition of calcium and phosphorus in the tibia. Wang et al [123] reported that lower zinc content in the diet led to a reduction in bone length, bone density and bone integrity, and calcium and phosphorus are the main components of bones. Similarly, Loveridge [112] stated that trace minerals are an important component in bone formation and development.

Blood parameters (total protein, albumin) were not sig nificantly influenced by the dose or form of zinc. Similarly, zinc addition in broiler diets does not improve serum parameters, including the protein concentration [124,125]. In contrast, Feng et al [20] reported that adding a diet with zinc increased the serum total protein concentrations. Zinc is related to blood protein levels because it is related to the metabolic activity of nutrients in the body because zinc supports enzymatic and physiological processes in the body, including the process of nutrient digestion in the digestive tract. Moreover, blood protein levels are associated with the time needed to digest nutrients in the digestive tract, and zinc is associated with nutritional digestive activity [116,126]. Walker et al [127] stated that health status could be considered from various aspects, including the protein content. Plasma proteins include albumin, globulin and fibrinogen, and they are involved in maintaining osmotic pressure, providing an amino acid source for tissues, transporting nutrients to cells and waste products to secretory organs, and maintaining the acid-base balance (buffer). Albumin has the ability to bind various ligands and is responsible for 80% of the osmotic pressure. Maggini et al [10] stated that zinc plays an important role in protein and nucleic acid synthesis because it is an important part of the enzymes involved their synthesis. Although significant differences were not observed, the different zinc forms increased the values of total protein and albumin compared with those in the control group.

In the current study, the addition of zinc to broiler feed had a positive impact on broiler production performance and could increase the ADG and the efficiency of feed use, which was based on a decrease in the FCR. Zinc also improved the broiler carcass quality by reducing the percentage of abdominal fat. In this study, the addition of zinc did not have a negative impact on bone mineral deposition, indicating that the addition of zinc did not have an antagonistic effect on calcium and phosphorus minerals. Accordingly, the current study can be used as a reference for broiler farmers to improve their broiler performance. Thereby, hopely the broiler farmers can get more economic benefits addition. The optimum addition zinc dose to broiler diet which affected the best ADG and feeds conversion ratio were 93.37 and 75.72 mg/kg, respectively, which will result in the best broiler production performance.

## CONCLUSION

This study discovers the positive impact of zinc addition on immunity and production performance of broilers through a comprehensive meta-analysis study. The optimum dose that produces the best pancreatic MT and reduced stress indicated by H/L ratio were 90.63 mg/kg and 106 mg/kg, respectively, which will perform the best broiler immunity. Meanwhile, the optimum addition zinc dose to broiler diet which affected the best ADG and FCR were 93.37 and 75.72 mg/kg, respectively, which will result in the best broiler production performance. Since the use of antibiotics in feed has been banned in many countries, adding zinc to broiler feed can help to increase broiler immunity.

## Figures and Tables

**Table 1:** Studies included in the meta-analysis of zinc addition on immune response and performance of broiler

No	References	Period (d)	Form of zinc	Dose (mg/kg)
1	Dozier et al [13]	0–17	Zinc sulphate; Availa zinc (organic)	40–120
2	Ao et al [14]	0–42	Zinc sulphate; Bioplex zinc (Zinc proteinate)	0–40
3	Aoyagi and Baker [15]	8–22	Zinc sulphate; Zinc lysine	0–8
4	Bartlett and Smith [16]	0–49	Zinc polyamino acid complex	34–181
5	Bafundo et al [17]	8–22	Zinc monocarbonate;	0–52
6	Cao et al [6618]	0–6	Zinc acetate; Zinc proteinate, Zinc methionine	0–90
7	Chan et al [4]	0–35	Zinc inorganic (ND)	0–60
8	Aksu et al [19]	0–42	Zinc sulphate	13–40
9	Feng et al [20]	0–42	Zinc glycine; Zinc sulphate	0–120
10	Huang et al [21]	0–21	Zinc sulphate	0–140
11	Dibaiee-nia et al [22]	0–21	Zinc oxide	0–100
12	Rao et al [23]	0–21	Bioplex zinc (Zinc proteinate)	0–40
13	Stahl et al [24]	0–21	Zinc carbonate	37–119
14	Sunder et al [25]	0–28	Zinc sulphate	0–160
15	Vieira et al [26]	0–41	Zinc sulphate; Zinc methionine	0–100
16	Jarosz et al [27]	0–42	Zinc sulphate; Zinc glycinate	100
17	Hudson et al [28]	0–17	Zinc sulphate; Zinc amino acid complex	160
18	Kwiecien et al [29]	0–42	Zinc oxide; Zinc glycinate	0–100
19	Li et al [30]	3–14	Zinc sulphate; Zinc tribasic sulphate	0–90
20	Yan et al [31]	0–42	Zinc sulphate	0–80
21	Kakhki et al [32]	0–35	Zinc methionine	0–120
22	Azad et al [33]	0–28	Zinc sulphate; Zinc methionine; Zinc-enriched yeast	0–80
23	Liao et al [34]	22–42	Zinc sulphate	0–140
24	Moghaddam and Jahanian [35]	0–42	Zinc sulphate; Zinc methionine; Zinc oxide	40
25	Sahoo et al [36]	0–42	Zinc sulphate; Zinc methionine	0–15
26	Chitithoti et al [37]	0–42	Zinc sulphate; Zinc methionine	0–80
27	Donmez et al [38]	0–45	Zinc sulphate	0–125
28	Roy et al [39]	0–35	Zinc sulphate	0–15
29	Sunder et al [40]	0–35	Commercial organic zinc (ND)	4–160
30	Ma et al [41]	0–21	Zinc glycinate	0–120
31	Mohanna and Nys [42]	5–21	Zinc sulphate; Zinc methionine	0–40
32	Pimentel et al [43]	0–28	Zinc oxide	8–88
33	Yang et al [44]	0–42	Inorganic zinc (as conventional inorganic sulphate salts)	0–120
34	Badawi et al [45]	0–42	Zinc sulphate ; Zinc methionine	0–40
35	Sajadifar [46]	0–42	Zinc sulphate	40–200
36	Yogesh et al [47]	0–42	Zinc sulphate; Zinc oxide; Zinc propionate	40–100
37	Kulkarni et al [6]	0–42	Zinc oxide	0–120
38	Ibrahim et al [48]	0–42	Zinc oxide; Zinc methionine	0–50
39	Lai et al [49]	0–42	Zinc oxide	0–60
40	Ivanisinova et al [50]	0–35	Zinc sulphate; Zinc-glycine, Zinc propionate	120
41	Ezzati et al [51]	0–42	Zinc sulphate	0–75

**Table 2:** Regression equations on the influence of zinc doses (in mg/kg of diet) on immune response of broilers

Response parameter	Unit	Model	N	Parameter estimates				Model estimates	
	
				Intercept	SE intercept	Slope	SE slope	p-value	AIC1)
Lymphoid organ weight									
Thymus	% BW	L	43	0.31	0.032	0.00026	0.00019	0.172	−81.8
Bursa fabricious	% BW	L	49	0.25	0.054	0.00013	0.00015	0.38	−87.7
Spleen	% BW	L	64	0.22	0.091	−0.00004	0.00015	0.81	−114
Liver	% BW	Q	27	2.14	0.265	−0.00472	0.001799	0.016	-
						0.000022	9.013E-6	0.0254	27.5
Haematologys									
Haemoglobin	g/dL	L	10	8.11	0.76	−0.00058	0.0035	0.872	27.1
Hematocryt	%	L	10	28.3	1.31	−0.017	0.008	0.083	41.1
H/L ratio	%/%	Q	18	0.585	0.0641	−0.00383	0.000851	0.0006	-
						0.000018	5.63E-6	0.0075	−8.7
Metallothionein									
Pancreas MT	RQ	Q	26	36	3.68	0.629	0.0765	<0.0001	-
						−0.00347	0.000611	<0.0001	173
Antioxidant activity									
SOD on meat	U/mg protein	L	10	53.5	2.84	0.043	0.053	0.444	60.7
SOD on liver	U/mg protein	L	21	161	21.9	0.23	0.17	0.198	211
MDA on liver	nmol/mL	L	15	4.31	0.670	−0.0055	0.003	0.097	39.5
Immunoglobulin (Ig) and complement (C)									
IgA	g/L	L	12	0.0406	0.014	0.000048	0.000177	0.792	−29
IgG	g/L	L	12	0.0484	0.010	0.000054	0.000124	0.671	−36.1
IgM	g/L	L	12	0.0442	0.011	0.000111	0.000137	0.437	−34.0
C3	g/L	L	12	0.0372	0.009	0.000107	0.000115	0.371	−37.6
C4	g/L	L	12	0.0284	0.010	0.000025	0.000125	0.848	−35.9

SE, standard error; AIC, akaike information criterion; BW, body weight; L, linear; Q, quadratic; H/L, heterophil/lymphocyte; MT, metallothionein; RQ, relative quantities of MT mRNA to β-actin mRNA in which RQ calculated by using this formula 2−ΔΔCt (Ct = threshold cycle); SOD, superoxide dismutase; MDA, malonaldehyde.

1) AIC is an estimator of the relative quality of statistical models for a given set of data.

**Table 3:** Influence of zinc forms on immune response of broilers

Response parameter	Unit	N	Zinc form			p-value

			Control	Organic	Inorganic	
Dose average	mg/kg		0	62.83	62.89	
Lymphoid organ weight						
Thymus weight	% BW	43	0.284	0.333	0.332	0.227
Bursa fabricious weight	% BW	49	0.228	0.263	0.272	0.248
Spleen weight	% BW	64	0.198	0.229	0.229	0.386
Liver weight	% BW	27	2.17^a^	2.00^ab^	1.90^b^	0.094
Haematology						
Haemoglobin	g/dL	10	8.13	nd	8.05	0.756
Hematocryt	%	10	27.9	nd	27.1	0.250
H/L ratio	%/%	18	0.60^b^	0.43^a^	0.46^a^	0.016
Metallothionein						
Pancreas MT	RQ	14	1.00^a^	nd	2.08^b^	0.002
Antioxidant activity						
SOD on meat	U/mg protein	10	46.5^a^	58.1^b^	52.9^ab^	0.009
SOD on liver	U/mg protein	21	158	197	176	0.312
MDA on liver	nmol/mL	15	4.28	3.86	4.01	0.546
Immunoglobulin (Ig) and complement (C)						
IgA	g/L	12	0.040	0.044	0.044	0.977
IgG	g/L	12	0.045	0.054	0.051	0.861
IgM	g/L	12	0.041	0.054	0.054	0.776
C3	g/L	12	0.035	0.046	0.049	0.714
C4	g/L	12	0.028	0.031	0.029	0.975

BW, body weight; nd, no available data; H/L, heterophil/lymphocyte; MT, Metallothionein; RQ, relative quantities of MT mRNA to β-actin mRNA in which RQ calculated by using this formula 2−ΔΔCt (Ct=threshold cycle); SOD, superoxide dismutase; MDA, malonaldehyde.

a,b Different superscripts within the same row are significantly different at p<0.05.

**Table 4:** Regression equations on the influence of zinc doses (in mg/kg of diet) on production performance, zinc concentration in tissue and bone, deposition of calcium and phosphorous in tibia of broilers

Response parameter	Unit	Model	N	Parameter estimates				Model estimates	
	
				Intercept	SE intercept	Slope	SE slope	p-value	AIC1)
Production performance									
Average daily gain	g/bird/d	Q	195	39.5	3.48	0.0691	0.0260	0.008	-
						−0.00037	0.000149	0.0131	1,355
Feed conversion ratio	g feed/g gain	Q	154	1.75	0.0546	−0.00083	0.000359	0.0223	-
						5.48E-6	1.98E-6	0.0060	−225
Mortality	%	L	12	0.30	0.962	0.0085	0.0085	0.344	51.6
Average daily intake	g/bird/d	L	174	71.1	6.66	0.018	0.014	0.21	1,310
Carcass percentage	%	L	14	68.7	2.66	0.0073	0.0120	0.552	69.1
Abdominal fat	%	L	11	1.01	0.219	−0.0027	0.00179	0.166	16.4
Zinc concentration									
Plasma	μg/mL	Q	76	1.35	0.215	0.010	0.00166	<0.0001	-
						−0.00004	8.56E-6	<0.0001	58.9
Tibia	mg/kg ash basis	Q	96	248	26.6	2.82	0.387	<0.0001	-
						−0.0121	0.00227	<0.0001	1,091
Liver	μg/g dry matter	L	27	2.01	0.25	−0.00056	0.00057	0.339	11.4
Meat	μg/g	Q	20	24.3	3.15	0.157	0.051	0.0083	-
						−0.00124	0.000482	0.0219	113
Minerals on Tibia									
Calcium	%	L	21	32.1	4.42	0.018	0.010	0.08	115
Phosphorous	%	L	18	15.3	0.99	0.0031	0.0044	0.496	64.1
Blood parameters									
Total of protein	g/L	L	12	28.6	1.46	0.023	0.017	0.215	62.9
Albumin	g/L	L	12	13.7	1.03	0.004	0.012	0.756	56.1

SE, standard error; AIC, akaike information criterion; Q, quadratic; L, linear.

1) AIC is an estimator of the relative quality of statistical models for a given set of data (smaller is better).

**Table 5:** Influence of zinc forms on production performance, zinc concentration in tissue and bone, deposition of calcium and phosphorous in tibia of broilers

Response parameter	Unit	N	Zinc form			p-value

			Control	Organic	Inorganic	
Dose average	mg/kg		0	62.83	62.89	
Production performance						
Average daily gain	g/bird/d	195	39.2	42.2	41.4	0.222
Feed conversion ratio	g feed/g gain	154	1.78^b^	1.72^a^	1.73^a^	0.017
Mortality	%	12	0	1.28	1.30	0.707
Average daily intake	g/bird/d	174	70.4	72.6	72.3	0.56
Carcass	%	14	68.1	69.4	69.5	0.448
Abdominal fat	%	11	1.18^a^	0.98^ab^	0.68^b^	0.034
Zinc concentration						
Plasma	μg/mL	76	1.31^b^	1.65^a^	1.88^a^	<0.0001
Tibia	mg/kg ash basis	96	230^b^	343^a^	369^a^	<0.0001
Liver	μg/g dry matter	29	87.6	97.4	96.4	0.352
Meat	μg/g	20	24.5^a^	27.5^b^	26.2^ab^	0.077
Minerals on Tibia						
Calcium	%	21	30.5^a^	33.7^b^	33.5^b^	0.023
Phosphorous	%	18	14.7^a^	16.6^c^	15.2^b^	<0.0001
Blood parameters						
Serum total of protein	g/L	12	27.5	30.8	30.4	0.363
Albumin	g/L	12	13.4	14.3	13.8	0.857

a–c Different superscripts within the same row are significantly different at p<0.05.
